# Quiescent-Interval Single-Shot Magnetic Resonance Angiography May Outperform Carbon-Dioxide Digital Subtraction Angiography in Chronic Lower Extremity Peripheral Arterial Disease

**DOI:** 10.3390/jcm11154485

**Published:** 2022-08-01

**Authors:** Judit Csőre, Ferenc Imre Suhai, Marcell Gyánó, Ákos András Pataki, Georgina Juhász, Milán Vecsey-Nagy, Dániel Pál, Daniele Mariastefano Fontanini, Ákos Bérczi, Csaba Csobay-Novák

**Affiliations:** 1 Heart and Vascular Center, Semmelweis University, 68 Városmajor Street, 1122 Budapest, Hungary; csore.judit@med.semmelweis-univ.hu (J.C.); suhai.ferenc.imre@gmail.com (F.I.S.); marcellgyanostb@gmail.com (M.G.); akospataki@hotmail.com (Á.A.P.); ginijs63@gmail.com (G.J.); vnagymilan@gmail.com (M.V.-N.); paldani91@gmail.com (D.P.); fontanini.med@gmail.com (D.M.F.); bercziak@gmail.com (Á.B.); 2 Department of Interventional Radiology, Semmelweis University, 68 Városmajor Street, 1122 Budapest, Hungary

**Keywords:** carbon dioxide, digital subtraction angiography, magnetic resonance angiography, peripheral arterial disease, renal insufficiency

## Abstract

Nephroprotective imaging in peripheral arterial disease (PAD) is often crucial. We compared the diagnostic performance of non-contrast Quiescent-interval single-shot magnetic resonance angiography (QISS MRA) and carbon-dioxide digital subtraction angiography (CO2 DSA) in chronic lower extremity PAD patients. A 19-segment lower extremity arterial model was used to assess the degree of stenosis (none, <50%, 50–70%, >70%) and the image quality (5-point Likert scale: 1-non-diagnostic, 5-excellent image quality). Intra-class correlation coefficient (ICC) was calculated for inter-rater reliability. Diagnostic accuracy and interpretability were evaluated using CO2 DSA as a reference standard. 523 segments were evaluated in 28 patients (11 male, mean age: 71 ± 9 years). Median and interquartile range of subjective image quality parameters for QISS MRA were significantly better compared to CO2 DSA for all regions: (aortoiliac: 4 [4–5] vs. 3 [3–4]; femoropopliteal: 4 [4–5] vs. 4 [3–4]; tibioperoneal: 4 [3–5] vs. 3 [2–3]; all regions: 4 [4–5] vs. 3 [3–4], all *p* < 0.001). QISS MRA out-performed CO2 DSA regarding interpretability (98.3% vs. 86.0%, *p* < 0.001). Diagnostic accuracy parameters of QISS MRA for the detection of obstructive luminal stenosis (70%<) as compared to CO2 DSA were as follows: sensitivity 82.6%, specificity 96.9%, positive predictive value 89.1%, negative predictive value 94.8%. Regarding the degree of stenosis, interobserver variability for all regions was 0.97 for QISS MRA and 0.82 for CO2 DSA. QISS MRA proved to be superior to CO2 DSA regarding subjective image quality and interpretability for the imaging of chronic lower extremity PAD.

## 1. Introduction

Peripheral arterial disease (PAD) affects almost 200 million people worldwide with an increasing incidence of 13–29% over the past decade [1,2]. Mortality is substantially increased in the affected patient population with a 40% chance of dying within 10 years and a six-fold higher chance of cardiovascular death [3]. Patients with diabetes and consequent renal failure are particularly prone to PAD [3,4], thus the use of nephroprotective imaging is crucial in the diagnosis. Carbon-dioxide digital subtraction angiography (CO2 DSA) is a well-established imaging tool in lower extremity PAD [5,6] and is considered as a reference standard due to its great spatial resolution. Being an invasive modality, however, it may cause complications associated with either the arterial puncture or the use of ionizing radiation [7,8,9]. The main threat of using CO2 as an intraarterial contrast agent is air embolism, which may result in bowel ischemia or stroke [10,11]. Therefore, non-invasive techniques such as contrast-enhanced magnetic resonance angiography (CEMRA) and computed tomography angiography (CTA) are gaining popularity for the imaging of lower extremity arterial disease in patients with normal renal function [4,12,13,14]. However, concerns have been raised for both modalities regarding their use in patients with impaired renal function: contrast-induced nephropathy (CIN) with iodinated contrast agents and nephrogenic systemic fibrosis (NSF) with gadolinium-based agents [15,16]. With Doppler ultrasound (US), we can detect the presence of occlusion and significant stenosis without contrast material or radiation exposure, and pulse-wave Doppler US can also show the exact flow parameters. It is a well-applicable method for follow-up, but imaging of the entire lower extremity vasculature is very time-consuming and it is highly affected by the operator’s experience [17]. Its use in complex lower extremity PAD—especially with severe atherosclerotic changes in the crural segments—is therefore not widespread. Quiescent-interval single-shot (QISS) MRA is a non-contrast protocol suitable for lower extremity arterial imaging [18,19,20,21,22,23,24,25,26,27].

In the present research, we compared the reproducibility, reliability, and diagnostic accuracy of QISS MRA and CO2 DSA in the imaging of chronic lower extremity arterial disease.

## 2. Materials and Methods

### 2.1. Study Design and Patient Population

Individuals with chronic lower extremity PAD who underwent CO2 DSA due to therapeutic decisions and treatment planning between June and December 2020 were enrolled to undergo additional QISS MRA imaging on the same day. Inclusion criteria of consecutive patients were as follows: (1) GFR was <30, or the patient did not consent to the administration of iodinated contrast agent, or there was a documented previous adverse reaction to iodinated contrast media; (2) informed consent to undergo an additional nonenhanced MRA examination; and (3) patient age >18 y. Exclusion criteria were: (1) patient age <18 y; and (2) patients with known contraindication to MRA. Indication of lower extremity angiography was at the discretion of the primary physician (vascular surgeon or angiologist) and was independent from this current study. In all cases, the investigators were blinded to the reports and the clinical findings of the patient. All patients provided informed consent. This single-center study was approved by the local and national ethical committees (registration number: OGYEI/7984/2020). All procedures were carried out in accordance with the Declaration of Helsinki.

### 2.2. CO_2_ DSA and QISS MRA Technical Specifications

All patients underwent diagnostic lower extremity CO2 DSA via transradial access, which was considered the reference standard for our study. Four-French pigtail catheters were used for the examinations. Non-selective contrast injection was used as standard with no table tilting. The use of selective injection was at the discretion of the radiologist and was only performed if non-selective images were insufficient regarding treatment planning. An automated injector (Angiodroid, Angiodroid SRL, Bologna, Italy) was used for CO2 delivery. Images were recorded with a modified CO2 DSA protocol (Siemens Evenflow, 3–4 frames per second (FPS) instead of 7.5 (official preset) with 60 mL injection volume at 500 Hgmm injection pressure regardless of region).

For QISS MRA, all tests were performed on a 1.5T MR scanner (MAGNETOM Aera, Siemens Healthineers, Erlangen, Germany). Feet first, supine patient positioning was used. We used a 36-element peripheral angiographic array coil system on lower extremities supplemented with two 18-element torso coils in the abdominal and pelvic region. QISS MRA scans were acquired in transversal plane under fat saturation and venous suppression. All measurements were performed under electrocardiographic gating in free breathing in the lower extremity and in breathold in the abdominal region. From the acquired transversal plane images, three dimensional (3D) rotating coronal plane maximum intensity projection (MIP) reconstructions were also obtained for all patients.

Regarding technical parameters, we used the same settings as Arendt et al. [18].

### 2.3. Image Analysis

Image processing for CO2 DSA and QISS MRA was performed on two different workstations according to the method (CO2 DSA: Leonardo; Siemens Healthcare, Erlangen, Germany; and QISS MRA: IMPAX EE, Agfa HealthCare, Bonn, Germany). For both methods, we have tried to achieve the best image quality by using optimal post-processing.

All CO2 DSA and QISS MRA images were evaluated by two radiologists with expertise of ten and four years in cardiovascular imaging independently during separate sessions.

Image quality and stenosis grading was scored for the following 19 segments: 1—aorta, 2 and 3—bilateral common iliac artery, 4 and 5—bilateral external iliac artery, 6 and 7—bilateral common femoral artery, 8 and 9—bilateral superficial femoral artery and popliteal artery, 10 and 11—bilateral deep femoral artery, 12 and 13—bilateral tibioperoneal trunk, 14 and 15—bilateral anterior tibial artery, 16 and 17—bilateral posterior tibial artery, 18 and 19—bilateral fibular artery. Segments that were identified as not imaged or just partially imaged by at least one reviewer were excluded from the analysis—a total of eight segments for QISS MRA (four aorta, four common iliac artery) and one segment for CO2 DSA (anterior tibial artery) were excluded due to partial imaging. A total of 523 segments were included in the final evaluation.

Image quality was analyzed according to a 5-point Likert scale on each segment: 1—non-diagnostic, image quality inadequate for diagnosis; 2—fair, image quality marginally acceptable for diagnosis; 3—moderate, image quality acceptable for diagnosis; 4—good, image quality adequate for confident diagnosis and 5—excellent, excellent image quality providing a highly confident diagnosis. The prevalence of major artifacts (susceptibility artifact, venous overlay, motion artifact and arrhythmia-related signal loss) has been listed separately in the assessment of QISS MRA examinations.

The classification of stenoses was given according to the scheme used in everyday practice, based on which we set up four categories of varying severity: no visible stenosis, degree of stenosis <50%, 50–70%, and >70%. The grading of stenosis was primarily performed visually, and in cases of questionable or borderline values, it was compared to an intact distal reference parameter. If there were multiple lesions in a segment, the most severe stenosis was given.

For the statistical analysis, the final values for both image quality and stenosis were decided by consensus of the two radiologists for the patients in question. We later divided the 523 segments into three prominent anatomical regions for the final evaluation—aortoiliac, femoropopliteal and tibioperoneal.

### 2.4. Statistical Analysis

Categorical data are presented as numbers (percentages), while continuous data are expressed as median and interquartile range (IQR). We used Wilcoxon matched-pair test for the comparison of Likert scores. The degree of inter-rater reproducibility was measured by using intraclass correlation coefficient (ICC). Levels of reliability were as follows: poor, ICC < 0.50; moderate, ICC = 0.5–0.74; good, ICC = 0.75–0.90; excellent, ICC > 0.90 [28]. Interpretability was also calculated both for QISS MRA and CO2 DSA using the proportion of non-diagnostic segments across all segments. Diagnostic accuracy parameters of QISS MRA for the detection of obstructive luminal stenosis (>70%) were calculated on a per-region level with CO2 DSA considered as the reference standard. A two-sided *p* < 0.05 was considered to be significant in all analyses. SPSS (version 25.0, Armonk, NY, USA) was used for all calculations.

## 3. Results

A total of 28 patients (11 male, mean age 71 ± 9 years) were analyzed. Further data of our patient population are shown in Table 1. In total, 523 segments were evaluated for subjective image quality in 164 prioritized regions (aortoiliac, femoropopliteal, tibioperoneal).

Following the consensus assessment, QISS MRA performed better than CO2 DSA in terms of image quality in all regions when comparing the two modalities (QISS MRA vs. CO2 DSA-good (4 [4–5]) vs. acceptable (3 [3–4]; *p* < 0.001), and was rated superior in the aortoiliac (good (4 [4–5]) vs. acceptable (3 [3–4]); *p* < 0.001), femoropopliteal (good (4 [4–5]) vs. good (4 [3–4]); *p* < 0.001) and tibioperoneal (good (4 [3–5]) vs. acceptable (3 [2–3]); *p* < 0.001) regions as well (Table 2. Per-artery comparison of interpretability between CO2 DSA and QISS MRA is shown in Table 3. As can be seen in the table, the proportion of non-diagnostic segments across all segments is 1.72% (9/523, *p* < 0.001) for QISS MRA and 13.96% for CO2 DSA (73/523; *p* < 0.001).

Average radiation exposure during CO2 DSA examinations was 5.99 ± 9.23 mGy*m^2.^ No complications occurred during the study in either modality.

### 3.1. Image Appearance and Artifacts

In two cases, the artifact caused by the implanted metal made the assessment limited—a screw fixation of the knee in one patient and a unilateral hip replacement in another patient caused susceptibility artifacts, which made the adjacent femoral and popliteal regions non-diagnostic.

Endovascular stent graft was placed in the iliac region in one patient; however, it did not significantly affect the diagnostic evaluation of the adjacent segments.

### 3.2. Stenosis Grading

QISS MRA also excelled in the classification of stenoses: inter-observer agreement showed better reproducibility for QISS MRA than CO2 DSA in all examined regions. The interobserver intraclass correlation coefficients (ICC) for the above regions for QISS MRA were 0.97 for all regions, 0.95 for aortoiliac, 0.97 for femoropopliteal, 0.97 for tibioperoneal, whereas ICC for CO2 DSA were 0.81, 0.80, 0.85 and 0.78 for the above regions, respectively.

The diagnostic accuracy parameters of QISS MRA using CO2 DSA as a reference standard are shown in Table 2 and Table 4.

## 4. Discussion

This study compared two nephroprotective imaging methods in the diagnosis of lower extremity PAD, CO2 DSA and QISS MRA. We had a slightly higher proportion of women (n = 17, 60.1%) in our patient population, which does not match international data [29] and may bias our results. Superior performance of QISS MRA was found regarding subjective image quality in all regions examined. For stenosis grading, the interobserver correlation coefficient for QISS MRA was particularly good, which confirms the great reproducibility of the method.

There is a lack of data regarding the comparison of the diagnostic performance of these techniques with only one retrospective study on 16 patients with chronic lower extremity PAD [18]. The image analysis of Arendt et al. was conducted on patients with readily available images of CO2 DSA and QISS MRA performed within 90 days. Our findings are in line with their conclusions, confirming the superiority of QISS MRA over CO2.

Noted, as given our current experience, both testing methods have their limitations.

**Figure 1:**
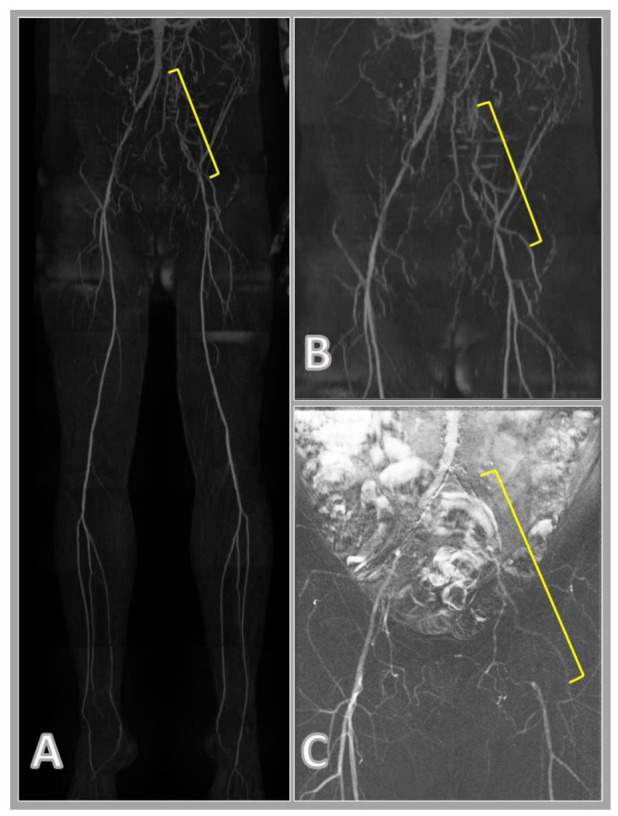
Comparison of quiescent-interval single-shot magnetic resonance angiography (QISS MRA (**A**,**B**); and carbon-dioxide digital subtraction angiography (CO2 DSA, (**C**)) images. QISS MRA images clearly show the occlusion of the left common and external iliac artery and the resulting collateralization (yellow bracket). Assessment of this region with CO2 DSA is severely compromised by bowel movements.

**Figure 2:**
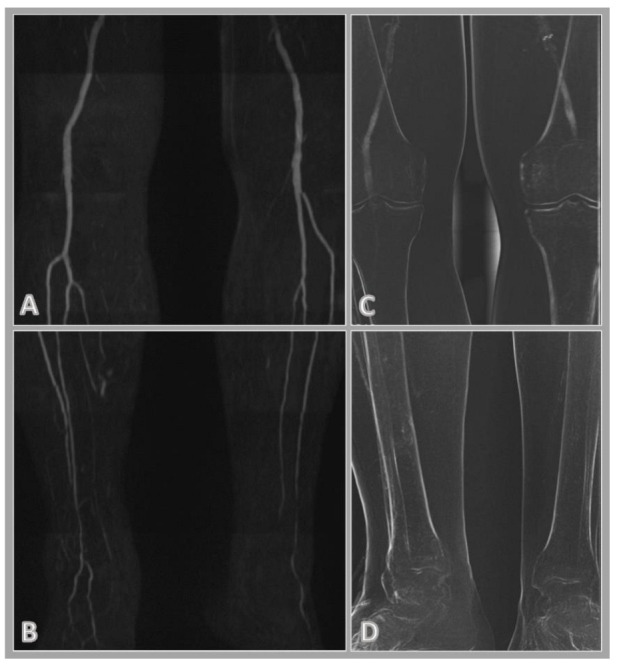
Corresponding quiescent-interval single-shot magnetic resonance angiography (QISS MRA; (**A**,**B**)) and carbon-dioxide digital subtraction angiography (CO2 DSA; (**C**,**D**)) images of the popliteal and crural region. CO2 DSA is compromised by the decreased arterial flow resulting in CO2 not reaching the crural vessels, whereas QISS MRA shows patent popliteal and peroneal arteries (**A**,**B**).

In the case of CO2 DSA, the assessment of the abdominopelvic region was hampered in several cases by the presence of artifacts caused by intestinal gases (Figure 1). The injected contrast material often breaks down into small bubbles, filling larger vessels with difficulty and not forming a continuous column in the lumen. To achieve good image quality, a high frame rate per second may be required, which significantly increases the radiation exposure. The use of CO2 is limited in patients with severe chronic obstructive pulmonary disease (COPD) or pulmonary hypertension [30] as well. A further limitation of the CO2 contrast material is that in the case of decreased blood flow, which is very common in patients with significant arterial stenosis or in patients in poor condition, it is more difficult to deliver contrast material to the distal region of the lower extremity. In the case of QISS MRA, decreased flow and the intestinal gases do not interfere with the evaluation of the images (Figure 2).

For QISS MRA, depending on the patient’s height, the abdominal aorta sometimes cannot be fully visualized with primer settings mentioned above. In this case, it may become necessary to image the missing region in one separate slab with an additional measurement. The artifact caused by stents implanted in the iliac region (cobalt chromium balloon expandable and nitinol self-expanding stents, or other, mixed variations) may make the accurate assessment of the vascular status even harder. Although these stents are usually safe to examine with MRI, for QISS MRA there is no data available on the evaluability of stents deployed in the iliac region. In our study, one patient has had a stent implanted in the past in another institution (Omnilink Elite, Abbott Laboratories, Abbott Park, Illinois), but in this case, the stent in the external iliac artery did not significantly affect diagnostic image quality. The evaluability of stents implanted in the pelvic and femoropopliteal region is most likely related to the material of the implanted stent. Varga-Szemes et al. claimed that different architecture of endovascular stents may explain why even patent stents of the same material can cause either signal loss or no interference [12].

Duration of the scan may cause additional problems: the whole examination (including the preparations) takes around 30 min. Considering that patients with poor conditions or rest pains may not tolerate this interval well, the examination can be particularly stressful, and the resulting motion artifacts may limit the diagnostic image quality. We also do not have sufficient information on studies in acute lower limb ischemia—the present and the previously mentioned [18] studies were performed in patients with chronic lower limb ischemia.

In addition, contraindications for QISS MRA may include anything that may contraindicate a general MR scan (intracranial aneurysm clips, claustrophobia, non-MR conditional implantable devices, etc.).

The 3D data set obtained with the QISS MRA has a great advantage and it can improve the evaluability of images, especially in the abdominal region. Rotating coronal MIP reconstruction, which can be easily generated from the image data, can still be a great help for the reader. Curved multiplanar reconstruction is also available on the basis of the 3D data set. The summation technique used for processing the image material of DSA examinations has a clear disadvantage in this region compared to QISS MRA regarding the intestinal gases and surrounding calcified structures as well. Compared with CO2 DSA, QISS MRA yields images with superior vessel contrast and no disturbing overlay from bone or calcified plaques.

The QISS MRA technique provided good image quality in the aortoiliac region, where breath-holding was applied to all patients during the non-contrast MRA scan, which may help to eliminate respiratory artifacts. In our experience, breath-holding did not significantly affect image quality, and the affected region was evaluable even in patients who were more difficult with cooperation. No previous literature data are available in this regard.

In the femoropopliteal region, both techniques had good image quality, and we found the smallest difference between the two techniques in this area. Generally, the stents implanted in the femoropopliteal region are almost exclusively made of nitinol—as in the aortoiliac region, there is no significant information available on their evaluability for the QISS MRA.

The difference in image quality was the strongest in the crural segments, where the image quality of QISS MRA was found to be significantly better than CO2 DSA in almost all cases. While the proportion of non-diagnostic segments for all regions in QISS MRA was negligible (9/523, 1.72%; *p* < 0.001), eight times as many segments were invaluable in CO2 DSA (73/523, 13.96%; *p* < 0.001). The difference was even more pronounced for the tibioperoneal region: for CO2 DSA, the number of non-diagnostic segments (63/223, 28.25%; *p* < 0.001) was 31.5 times higher than with QISS MRA (2/223, 0.89%; *p* < 0.001).

In the crural region, DSA with the use of iodinated contrast gives good image quality, but the diagnostic value of the test may be reduced by the use of CO2 contrast. A more selective use of CO2 contrast agent could improve the image quality but increase the complexity and invasiveness of the diagnostic procedure with extra radiation exposure and time. For all diagnostic imaging studies, the aim is to use the minimum invasiveness necessary to make therapeutic decisions. This is where the QISS MRA proved to have the best signal-to-noise ratio and the flow direction was perfect for the application of this measurement method in this region of interest.

Compared to alternative imaging methods, QISS MRA has both advantages and disadvantages as well [31,32].

In the case of non-invasive, non-contrast Doppler ultrasound, the quality of the examination is highly dependent on the experience of the examiner and the imaging of the entire lower limb takes a long time (up to two hours) [17]. If there is a high-grade stenosis or occlusion in the proximal part of the lower limb arterial system, the assessment of more distal vascular segments is very limited due to changes in flow parameters. Extensive calcification also complicates the evaluation. However, the method is well suited for the assessment of focal lesions, screening, and follow-up of vascular interventions. QISS MRA is an easy-to-use tool that can depict the entire lower limb vasculature in about 30 min, regardless of flow parameters.

CTA is an excellent and rapid diagnostic method, especially in the aortoiliac and femoropopliteal regions, but calcified plaque formation in the crural region can make the assessment of stenosis quite difficult. A great advantage is that detailed information on the characteristics of the plaque (calcified, non-calcified, partially calcified) can be obtained. With QISS MRA, calcified plaques are not visible but if necessary, some advanced MR techniques can also be used to detect vascular calcifications [33,34,35].

Contrast-enhanced MRA has good image quality but, like CTA, may not be suitable for patients with renal failure. The iodinated contrast agent DSA has an excellent spatial and temporal resolution, but we must also be aware of complications due to contrast administration and arterial puncture, which is not a problem with non-contrast, non-invasive QISS MRA. Considering the above advantages and disadvantages, it is up to the clinician and the radiologist to choose the most suitable diagnostic method for the particular patient.

### Study Limitations

Our study is a single-center, retrospective study with a relatively small number of patients. We had a slightly higher proportion of women in our patient population, which may bias our results.

We considered CO2 DSA as the reference standard, which has often proved to be of limited diagnostic value in the crural region—thus using iodinated contrast agent could have served as a better reference standard.

## 5. Conclusions

QISS at 1.5 Tesla has proven to be a more reproducible, reliable test method than CO2 DSA in the diagnosis of lower limb arterial stenosis. Thus, our results suggest that QISS MRA, if available, is a safe and reliable non-invasive diagnostic alternative in patients with chronic renal failure and in patients in whom gadolinium and iodinated contrast agents are contraindicated for various other reasons. Given the technique’s high accuracy, QISS provides a viable alternative to CTA, CEMRA, and DSA even in patients with normal renal function.

## Figures and Tables

**Table 1:** Characteristics of the patient population. Continuous variables are described as median and interquartile range (IQR), whereas categorical variables are represented as frequencies and percentages. (BMI: body mass index; eGFR: estimated glomerular filtration rate; KDOQI: Kidney Disease Outcomes Quality Initiative).

Parameters	Patients (n = 28)
*General parameters*	
Age (years)	
Median [IQR]	71 [8.8]
Female sex, N (%)	17 (60.7)
Height (cm)	
Median [IQR]	167 [14.5]
Weight (kg)	
Median [IQR]	79 [18.5]
*Atherosclerotic risk factors*	
BMI >25 kg/m^2^, N (%)	21 (75.0)
Smoking (current and former), N (%)	25 (89.3)
Hypertension, N (%)	17 (60.7)
Dyslipidemia, N (%)	7 (25.0)
Diabetes mellitus, N (%)	10 (35.7)
*Chronic renal insufficiency*	
eGFR (ml/min/1.73 m^2^)	
Median [IQR]	55 [36.9]
KDOQI stage II, N (%)	11 (39.3)
KDOQI stage III, N (%)	10 (35.7)
KDOQI stage IV, N (%)	6 (21.4)
*Peripheral arterial disease*	
Fontaine stage I, N (%)	0 (0.0)
Fontaine stage IIa, N (%)	1 (3.6)
Fontaine stage IIb, N (%)	15 (58.6)
Fontaine stage III, N (%)	2 (7.1)
Fontaine stage IV, N (%)	8 (28.6)
*Other comorbidities*	
Other peripheral arterial disease, N (%)	4 (14.3)
Coronary artery disease, N (%)	2 (7.1)
Atrial fibrillation, N (%)	4 (14.3)
Arrhythmia, N (%)	2 (7.1)

**Table 2:** Per-region assessment of subjective image quality and reproducibility for stenosis grading of CO2 DSA compared to QISS MRA. Values are expressed as median with interquartile ranges in brackets. CO2 DSA, carbon dioxide digital subtractive angiography. (CO2 DSA: carbon dioxide subtraction angiography, QISS MRA: quiescent-interval single-shot magnetic resonance angiography, ICC: intraclass correlation coefficient, CI: confidence interval).

	QISS MRA			CO2 DSA		
All regions	4 [4–5]	ICC: 0.97	[95% CI: 0.96–0.97] *p* < 0.001	3 [3–4]	ICC: 0.81	[95% CI: 0.73–0.86] *p* < 0.001
Aortoiliacal region	4 [4–5]	ICC: 0.95	[95% CI: 0.93–0.96] *p* < 0.001	3 [3–4]	ICC: 0.80	[95% CI: 0.63–0.90] *p* < 0.001
Femoropopliteal region	4 [4–5]	ICC: 0.97	[95% CI: 0.96–0.8] *p* < 0.001	4 [3–4]	ICC: 0.85	[95% CI: 0.75–0.91] *p* < 0.001
Tibioperoneal region	4 [3–5]	ICC: 0.97	[95% CI: 0.96–0.98] *p* < 0.001	3 [2–3]	ICC: 0.78	[95% CI: 0.67–0.87] *p* < 0.001

**Table 3:** Per-artery comparison of interpretability between CO2 DSA and QISS MRA. (CO2 DSA: carbon dioxide subtraction angiography, QISS MRA: quiescent-interval single-shot magnetic resonance angiography).

	QISS MRA	CO2 DSA	*p*-Value
ALL REGIONS, % (n/N)	98.3 (514/523)	86.0 (450/523)	*<0.001*
AORTOILIACAL REGION, % (n/N)	96.7 (128/132)	93.9 (124/132)	0.24
FEMOROPOPLITEAL REGION, % (n/N)	98.2 (165/168)	98.8 (166/168)	0.67
TIBIOPERONEAL REGION, % (n/N)	99.1 (221/223)	71.7 (160/223)	*<0.001*

**Table 4:** Diagnostic accuracy parameters of quiescent-interval single-shot magnetic resonance angiography (QISS MRA) for the detection of obstructive luminal stenosis (>70%), as compared to carbon-dioxide digital subtraction angiography (CI: confidence interval).

	QISS MRA
SENSITIVITY	82.6% [95% CI, 74.1–89.2%]
SPECIFICITY	96.9% [95% CI, 94.6–98.5%]
POSITIVE PREDICTIVE VALUE	89.1% [95% CI, 82.0–93.6%]
NEGATIVE PREDICTIVE VALUE	94.8% [95% CI, 92.4–96.5%]
DIAGNOSTIC ACCURACY	93.6% [95% CI, 91.0–95.6%]

## Data Availability

Not applicable.
